# CC chemokine receptors are prognostic indicators of gastric cancer and are associated with immune infiltration

**DOI:** 10.1186/s12920-023-01690-w

**Published:** 2024-01-02

**Authors:** Xinghe Liao, Yong Yang, Lihuan Wang, Zhiyuan Kong, Weiping Li

**Affiliations:** 1 Department of Integrated Therapy, Fudan University Shanghai Cancer Center, Shanghai, 200032 China; 2 Department of Oncology, Shanghai Medical College, Fudan University, Shanghai, 200032 China; 3 Department of General Surgery, the First Affiliated Hospital of Nanchang University, Nanchang, 330006 Jiangxi Province China; 4 Department of Radiology, the First people’s Hospital of Taicang City, Taicang Affiliated Hospital of Soochow University, Taicang City, 215400 Jiangsu Province China; 5 Department of Gastrointestinal Surgery, the First people’s Hospital of Taicang City, Taicang Affiliated Hospital of Soochow University, Taicang City, 215400 Jiangsu Province China

**Keywords:** CC chemokine receptors, Gastric cancer, Biomarker, Prognosis, Immune infiltrates

## Abstract

**Background:**

CC chemokine receptors are responsible for regulating the tumor microenvironment (TME) and participating in carcinogenesis and tumor advancement. However, no functional study has investigated CC chemokine receptors in gastric cancer (GC) prognosis, risk, immunotherapy, or other treatments.

**Methods:**

We conducted a bioinformatics analysis on GC data using online databases, including the Human Protein Atlas (HPA), Kaplan-Meier (KM) plotter, GeneMANIA, MethSurv, the University of ALabama at Birmingham CANcer (UALCAN) Data Analysis Portal, Gene Set Cancer Analysis (GSCA), cBioportal, and Tumor IMmune Estimation Resource (TIMER).

**Results:**

We noted that CC chemokine receptor expression correlated with survival in GC. CC chemokine receptor expression was also strongly linked to different tumor-infiltrating immune cells. Additionally, CC chemokine receptors were found to be broadly drug-resistant in GC.

**Conclusion:**

Our study identifed CC chemokine receptor expression helped in predicting the prognosis of patients diagnosed with GC. The expression level of the CC chemokine receptors was also positively related to multiple tumor-infiltrating lymphocytes (TILs). These findings provide evidence to monitor patients with GC using CC chemokine receptors, which can be used as an effective biomarker for predicting the disease prognosis and be regarded as a therapeutic target for modulating the tumor immune microenvironment.

## Introduction

Among cancers, GC is a common malignancy in China. GC ranks sixth and third in terms of cancer incidence and death, respectively [1, 2]. Currently, surgical resection is considered the main form of treatment for early-stage GC patients; however, most patients are inoperable at the time of diagnosis [3–5]. The GC patients show a low 5-year overall survival (OS) rate since a majority of these patients get diagnosed in their advanced stages of GC [6–8]. These issues have highlighted the need for identifying novel biomarkers for early diagnosis, prediction of metastatic progression, and prognosis of GC patients.

Furthermore, the infiltration of various immune cells in the TME is an important factor for determining the malignant tumor genesis, development, metastasis, and therapy resistance of cancer [9–11]. In existing studies receptor tyrosine kinases (RKTs) (e.g., EGFR, FGFR2, HER2, and MET), PD-L1, claudin 18.2 are frequently overexpressed [12].

CC chemokine receptors, or the beta chemokine receptors, belong to the G protein-linked receptor superfamily called the seven-transmembrane domain receptors [13]. These receptors are a type of membrane protein that can specifically bind to the CC chemokine family of the cytokines. According to the International Union of Immunological Societies (IUIS), which is a World Health Organization (WHO) Subcommittee on Chemokine Nomenclature, there are 10 CC chemokine receptors, namely CCR1-CCR10 [14, 15]. These CC chemokine receptors are involved in many biological activities, like recruiting immune cells, regulating leukocyte chemotaxis, tumorigenesis, inflammation, parenchymal remodeling, and cancer progression [16–20]. Several types of cells like immune cells, tumor cells, peripheral blood cells, and stromal cells, express CC chemokine receptors. These receptors assist in determining the composition of the tumor stroma and are associated with tumor growth, metastasis, and angiogenesis. Thus, they directly or indirectly influence the progression of cancer, therapeutic effects, and the resulting clinical outcome [21–24]. Predictive biomarkers are the mainstay of precision medicine.

Although several researchers have studied different types of CC chemokine receptors in the past and also described their expression and functions, it is unclear how the CC chemokine receptors work as targets and indicators in GC. In this study, we used publicly-accessible bioinformatics datasets to examine the survival and function of CC chemokine receptors in GC to determine its prognostic mechanism.

## Materials and methods

### Kaplan-Meier (KM) plotter

We used the KM plotter (http://www.kmplot.com) to examine the prognostic role of CC chemokine receptors in GC [25]. The researchers also calculated the Log-rank *P*-value and the hazard ratio (HR) at the 95% confidence interval (CI).

### Human protein atlas (HPA)

We used the immunohistochemical data obtained from the HPA repository (https://www.proteinatlas.org/) for determining the expression levels of the CC chemokine receptors in gastric tissues [26]. To analyze the relevant spatial protein expression patterns, immunohistochemically labeled tissue sections as well as the Single Cell Type in HPA data, which was based on the single-cell RNA sequence (scRNAseq) data derived from the Peripheral Blood Mononuclear Cells (PBMCs) and tissue samples of 25 patients, were used.

### Tumor IMmune Estimation Resource (TIMER)

We used the TIMER algorithm dataset (https://cistrome.shinyapps.io/timer/) to investigate the correlation between the CC chemokine receptor expression level in normal and GC patients [27]. The TIMER dataset includes information regarding 32 different cancer types and contains 10,897 samples derived from The Cancer Genome Atlas (TCGA). It also includes data related to the immune cells like CD8 + T cells, CD4 + T cells, macrophages, neutrophils, B cells, and dendritic cells.

## GeneMANIA

The protein-gene interactions, pathways, and functions of the CC chemokine receptors, along with all their related interaction were predicted with the help of GeneMANIA (http://www.genemania.org) [28].

### cBioPortal

The cBioPortal (http://cbioportal.org) is a simple online application that allows users to find multifaceted cancer genomic datasets and retrieve information from over 5000 tumor specimens from more than 20 cancer-related studies [29]. The cBioPortal dataset was utilized to investigate CC chemokine receptors’ mutation in GC. The frequency and the types of genomic alteration of CC chemokine receptors were investigated. The genomic changes in CC chemokine receptors included deep deletion, mRNA up-modulation, copy number amplification, and missense mutation with unclear significance, among others.

### The UALCAN Data Analysis Portal

UALCAN (http://ualcan.path.uab.edu/), which is an interactive web tool that helps in analyzing cancer data, was used to examine the correlation between the CC chemokine receptor expression level and DNA methylation status among GC patients [30].

### Gene Set Cancer Analysis

GSCA (http://bioinfo.life.hust.edu.cn/GSCA/#/) is an optimized version of GSCALite, which is a database that helps in searching, analyzing, and exploring the gene set cancer analysis associated with immune infiltration, mRNA expression, mutation, and drug resistance [31]. The GSCA combines the data corresponding to 10,000 multi-dimensional genomic data points for the 33 different cancer types described in the TCGA and more than 750 small molecule drug-related data derived from the Genomics of Drug Sensitivity in Cancer (GDSC) portal and the Cancer Therapeutics Response Portal (CTRP).

### MethSurv

We investigated the prognostic significance of the single CpG methylation status of CC chemokine receptors in the GC patients using MethSurv (https://biit.cs.ut.ee/methsurv/). It is a web platform that helps in survival analysis based on the CpG methylation pattern [32].

## Results

### mRNA expression levels of the CC chemokine receptors in GC patients

**Fig. 1:**
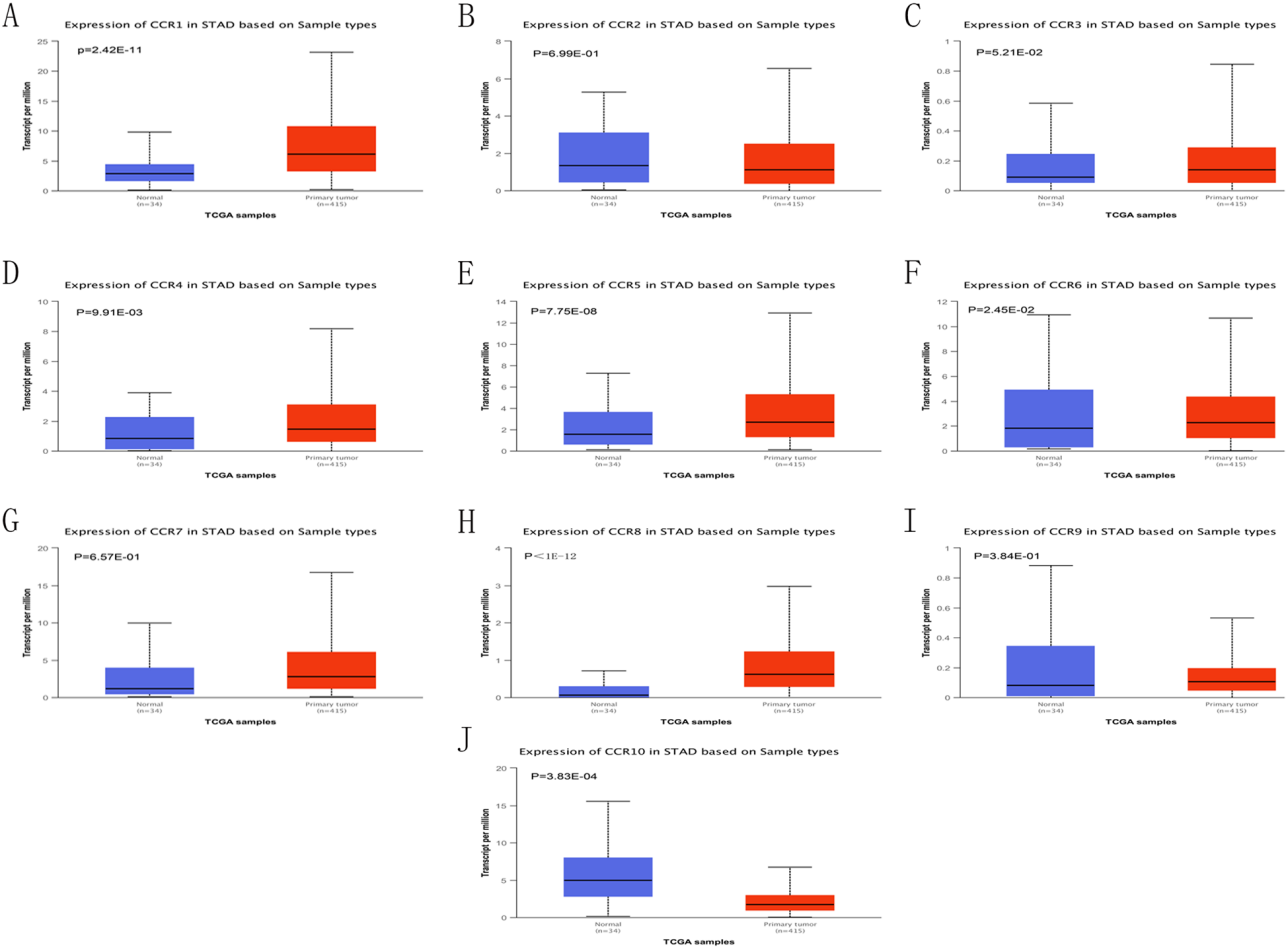
Differential expression of the CC chemokine receptors in differing disease states using UALCAN

We studied the differential expression of the CC chemokine receptors in GC and normal tissue samples using the UALCAN repository. The results indicated that CCR10 and CCR6 expression levels were significantly lowered in the GC tissues, whereas the CCR1, CCR4, CCR5, and CCR8 gene expression levels were significantly elevated in the GC tissues than in normal gastric tissues (*p* < 0.05). (Fig. 1).

**Fig. 2:**
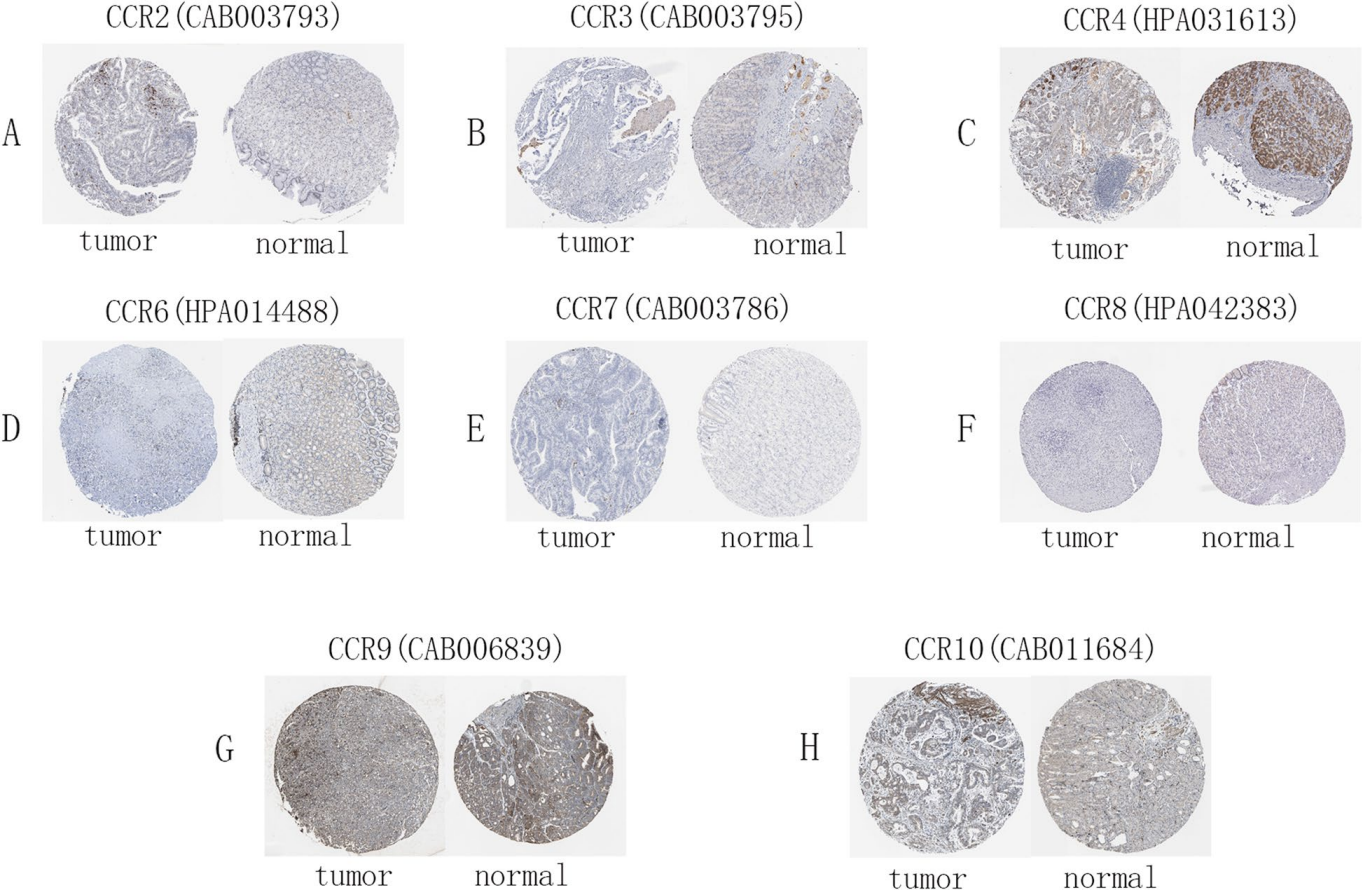
Immunohistochemistry images presenting the expression of the CC chemokine receptors in the GC tissues and normal tissues using HPA

The expression levels of the CCR2, CCR3, CCR4, CCR6, CCR7, CCR8, CCR9, and CCR10 proteins in STAD were also determined using HPA (Fig. 2). CCR1 and CCR5 were not found in HPA. We found that CCR4 and CCR8 stained more prominently in tumor tissues. CCR6 and CCR10 immunostaining was less pronounced in tumor tissues.

### Prognostic potential of CC chemokine receptors in GC

**Fig. 3:**
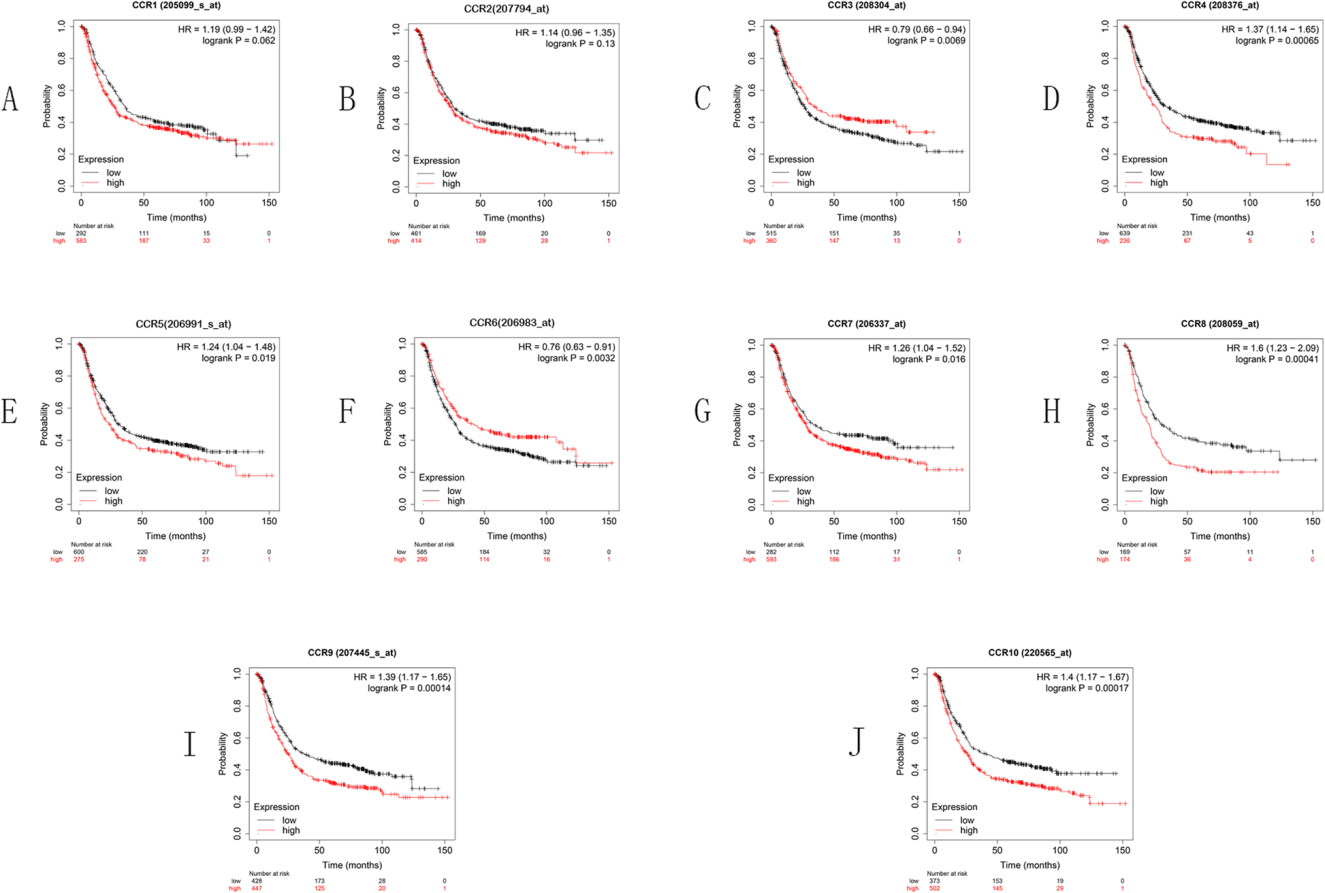
OS curves for the CC chemokine receptor expression in GC using the KM plotter

The OS curves for the expression of the CC chemokine receptor are shown in Fig. 3 for the KM plotter dataset, which is on the basis of the Affymetrix microarrays. The high expression levels of CCR4 (*p* = 0.00065), CCR5 (*p* = 0.019), CCR7 (*p* = 0.016), CCR8 (*p* = 0.00041), CCR9 (*p* = 0.00014), and CCR10 (*p* = 0.00017) indicate shorter OS, while the high expression levels of CCR3 (*p* = 0.0069) and CCR6 (*p* = 0.0032) indicate a longer OS in GC patients.

**Fig. 4:**
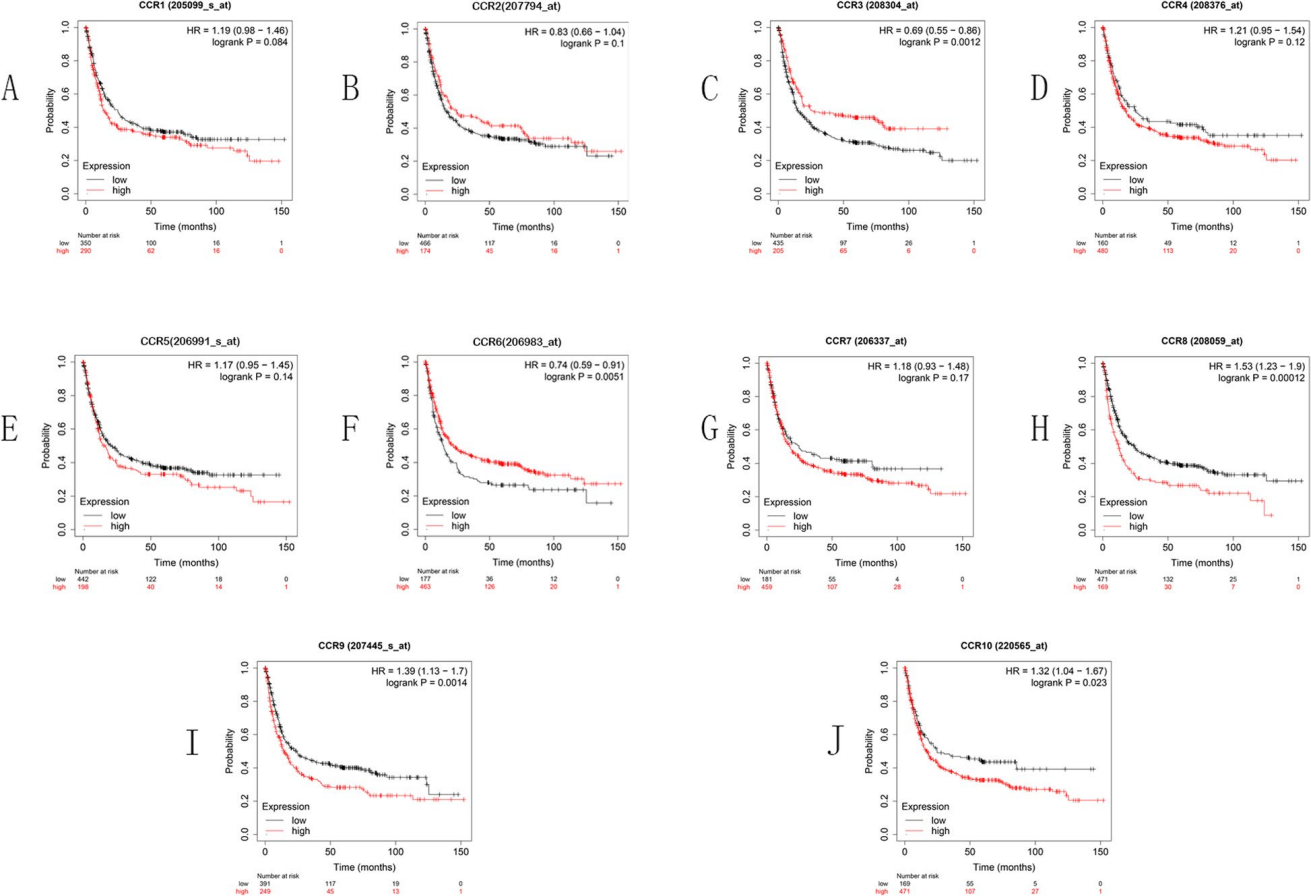
FPS curves for CC chemokine receptor expression in GC using the KM plotter

The first-progression survival (FPS) curves for the CC chemokine receptor expression in GC are presented in Fig. 4. The high expression levels of CCR8 (*p* = 0.00012), CCR9 (*p* = 0.0014), and CCR10 (*p* = 0.023) indicate shorter FPS, while high expression levels of CCR3 (*p* = 0.0012) and CCR6 (*p* = 0.0051) indicate longer FPS in GC cases.

**Fig. 5:**
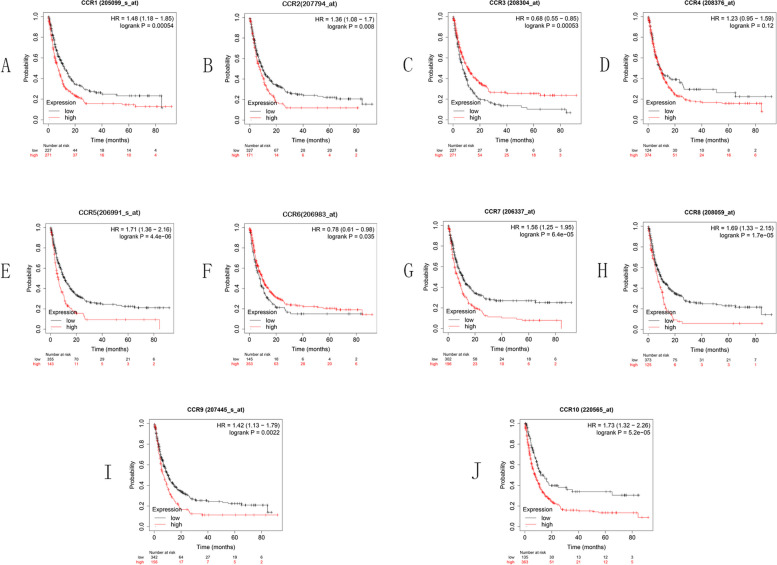
PPS curves for CC chemokine receptor expression in GC using the KM plotter

Post-progression survival (PPS) CC chemokine receptor expression in GC are presented in Fig. 5. The high expression levels of CCR1 (*p* = 0.00054), CCR2 (*p* = 0.008), CCR5 (*p* = 4.4e-06), CCR7 (6.4e-05), CCR8 (1.7e-05), CCR9 (*p* = 0.0022), and CCR10 (*p* = 5.2e-05) indicate shorter PPS, whereas the high expression levels of CCR3 (*p* = 0.00053) and CCR6 (*p* = 0.035) indicate longer PPS in GC cases.

### Correlation analysis between the DNA methylation status and CC chemokine receptor expression levels

**Fig. 6:**
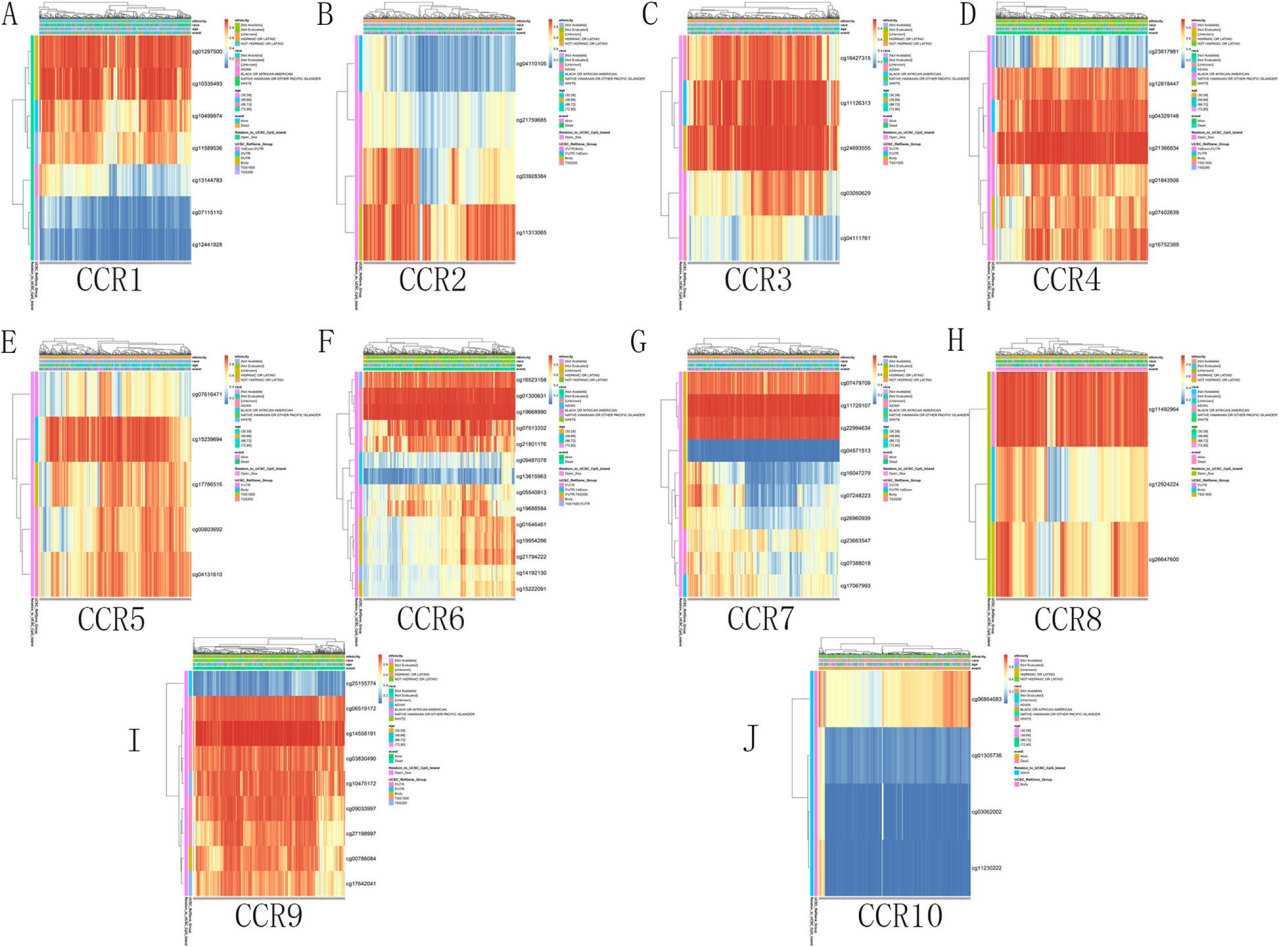
Heat maps of the DNA methylation expression levels of the CC chemokine receptor genes using MethSurv

We determined the predictive value of the DNA methylation status of the CC chemokine receptors in GC using MethSurv. Figure 6 presents the heat map of DNA methylation status in the CC chemokine receptors. The results showed that the cg10335493 gene coding for CCR1, cg11313065 gene coding for CCR2, cg24693555 gene coding for CCR3, cg21366834 gene coding for CCR4, cg15239694 gene coding for CCR5, cg19668990 gene coding for CCR6, cg11729107 gene coding for CCR7, cg11492964 gene coding for CCR8, cg14558191 gene coding for CCR9, and cg06864083 gene coding for CCR10 displayed the highest levels of DNA methylation. We found that 4 CpGs of CCR1, 1 CpG of CCR2, 1 CpG of CCR3, 4 CpGs of CCR4, 2 CpGs of CCR5, 2 CpGs of CCR6, 1 CpG of CCR7, 1 CpG of CCR8, 3 CpGs of CCR9 and 1 CpG of CCR10 were significantly associated with prognosis in GC patients (Table 1).

**Table 1:** The Prognostic Value of Single CpG of CC Chemokine Receptors in GC by MethSurv (P < 0.05)

Gene-CPG	HR	LR-test P-value
CCR1-5’UTR-Open_sea-cg10335493	0.692	0.033
CCR1-TSS200-Open_sea-cg07115110	1.668	0.0019
CCR1-TSS200-Open_sea-cg12441928	1.491	0.017
CCR1-TSS1500-Open_sea-cg12441928	0.718	0.044
CCR2-Body-Open_sea-cg11313065	0.663	0.037
CCR3-TSS1500-Open_sea-cg16427315	0.654	0.011
CCR4-Body-Open_sea-cg07402639	1.473	0.023
CCR4-TSS1500-Open_sea-cg16752389	1.77	0.0012
CCR4-TSS1500-Open_sea-cg21366834	1.394	0.043
CCR4-TSS200-Open_sea-cg23817981	1.449	0.024
CCR5-TSS200-Open_sea-cg04131610	0.689	0.036
CCR5-Body-Open_sea-cg15239694	0.522	0.00037
CCR6-5’UTR;TSS200-Open_sea-cg01646461	0.665	0.038
CCR6-5’UTR;1stExon-Open_sea-cg13615963	1.41	0.039
CCR7-Body-Open_sea-cg11729107	0.65	0.028
CCR8-5’UTR-Open_sea-cg11492964	0.687	0.025
CCR9-Body-Open_sea-cg00786084	0.614	0.0086
CCR9-TSS1500-Open_sea-cg09033997	0.647	0.0095
CCR9-TSS1500-Open_sea-cg27198997	0.646	0.0077
CCR10-Body-Island-cg03062002	1.396	0.043

### Correlation analysis between CC chemokine receptor expression level and the infiltrating immune cells

**Fig. 7:**
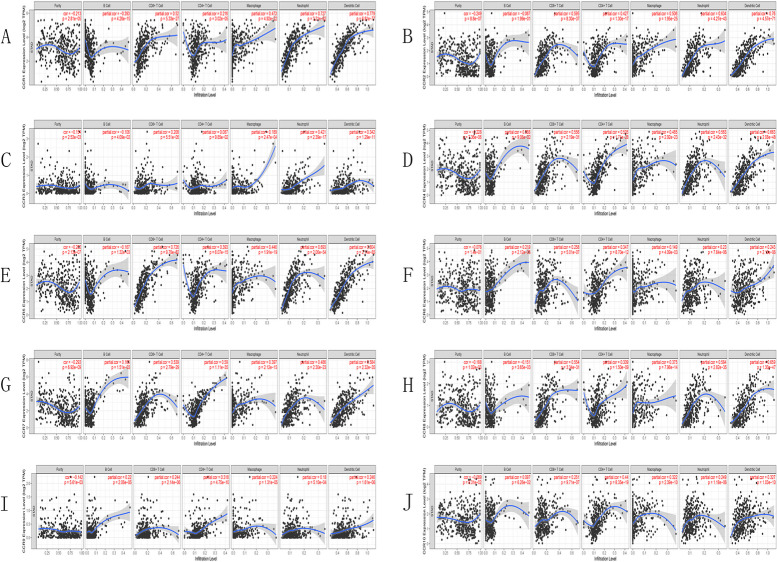
Relationship between the CC chemokine receptor expression levels and the immune cell infiltration level using TIMER

We investigated the relationships between CC chemokine receptor expressions and 6 types of infiltrating immune cells (neutrophils, macrophages, B cells, dendritic cells, CD4 T cells, and CD8 + T cells) with the aid of the TIMER database. The results are presented in Fig. 7. The results showed that the CC cytokine receptor expression level was positively correlated with the infiltration of 6 types of immune cells, except for CCR2 and CCR10 in B cells.

**Fig. 8:**
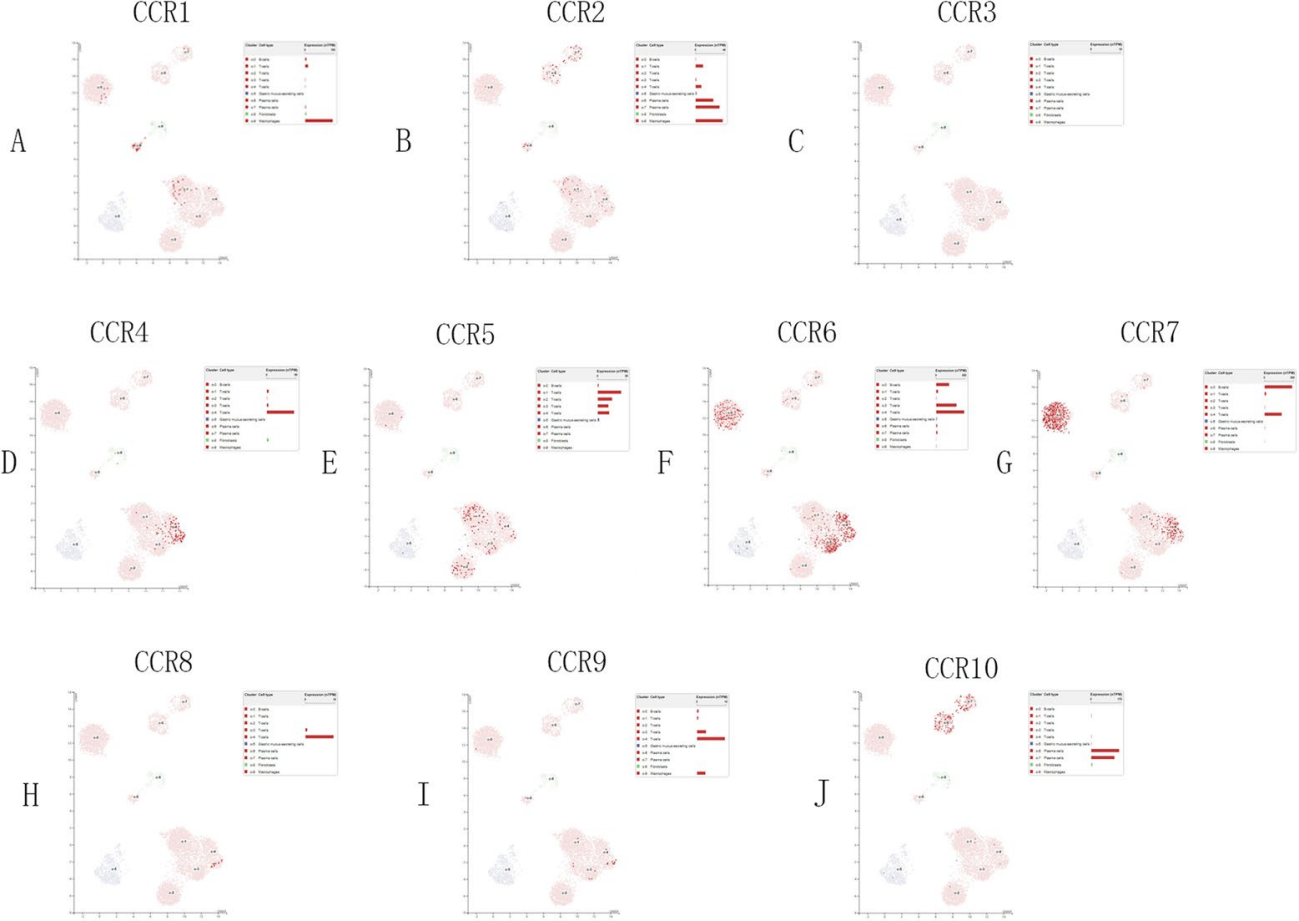
Relationship between the CC chemokine receptor expression levels and the tumor-infiltrating immune cells in GC using HPA

For determining the expression of the CC chemokine receptors in the tumor-infiltrating immune cells in GC, the researchers used the Single Cell Type in HPA; the results are presented in Fig. 8. Except for CCR3, the expression level of CCR1 was high in macrophages; CCR2 expression level was high in macrophages and plasma cells; CCR4, CCR5, and CCR8 expression levels were high in T cells; CCR6 and CCR7 expression levels were high in T cells and B cells; CCR9 expression level was high in macrophages and T cells, and CCR10 expression level was high in plasma cells.

### Genetic alterations and Gene Interaction analyses of the CC chemokine receptors in GC patients

**Fig. 9:**
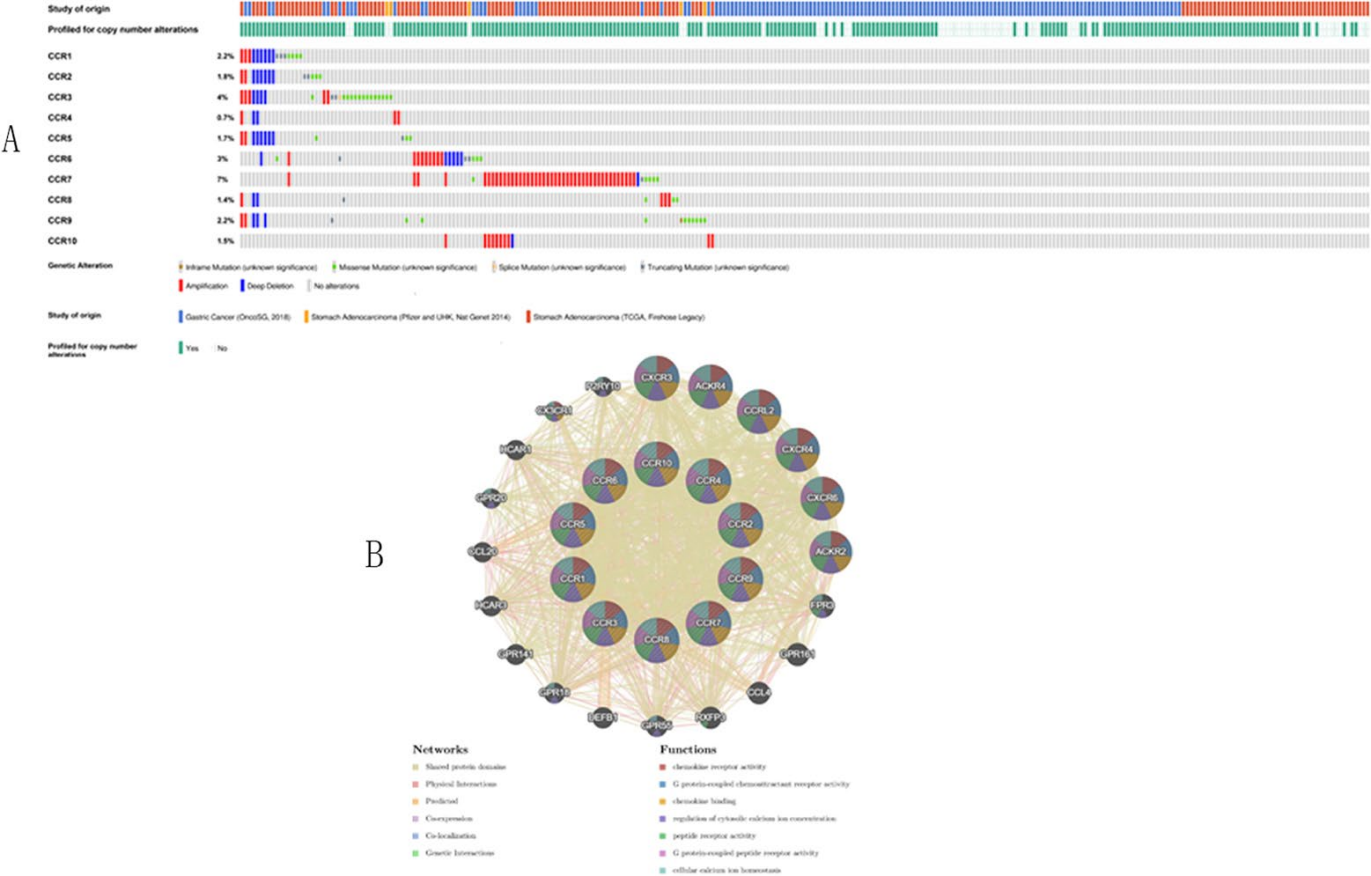
**A** CC chemokine receptor expression in GC from the cBioportal database. **B** The interactive analysis of CC chemokine receptors in the GC tissues using GeneMANIA databases

We also conducted a thorough examination of the molecular properties of the CC chemokine receptors. Firstly, cBioPortal was used to examine the genetic alterations of the CC chemokine receptors. The gene alterations of CCR1, CCR2, CCR3, CCR4, CCR5, CCR6, CCR7, CCR8, CCR9, and CCR10 were seen to be 2.2%, 1.8%, 4%, 0.7%, 1.7%, 3%, 7%, 1.4%, 2.2%, and 1.5% of the LUAD samples, respectively (Fig. 9A). Additionally, GeneMANIA results showed that the functions of the above CC chemokine receptors were correlated with the cytokine receptor activity, G-protein coupled receptor activity, G-protein coupled chemoattractant receptor activity, and the chemokine-mediated signaling pathways (Fig. 9B).

### Drug sensitivity of the CC chemokine receptors in GC patients

**Fig. 10:**
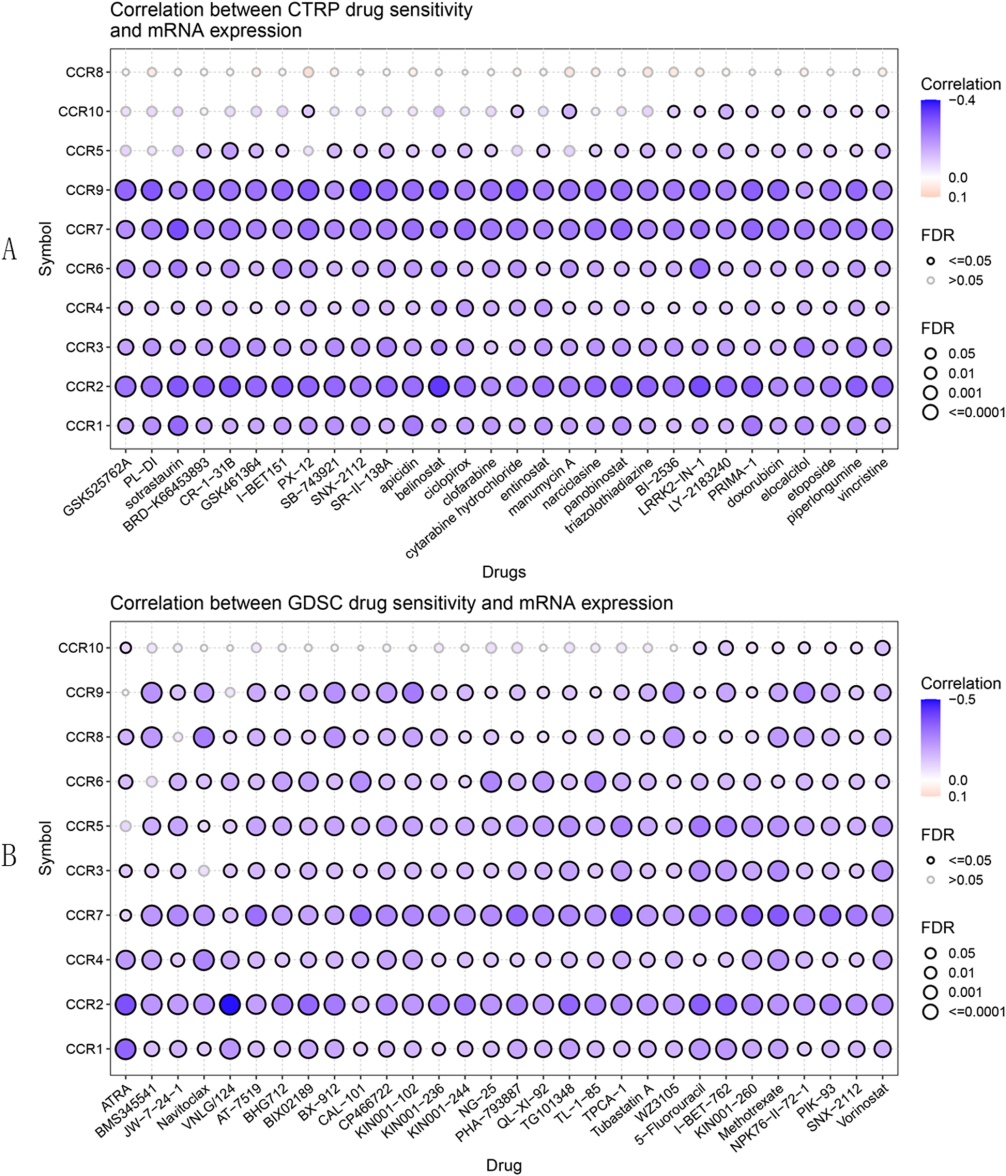
The drug sensitivity of CC chemokine receptors in GC. **A** Correlation between gene expression levels and sensitivity to CTRP drugs (Top 30). **B** Relationship between gene expression levels and sensitivity to GDSC drugs (Top 30)

Using GSCA, CC chemokine receptors were observed to exhibit resistance to multiple drugs (Fig. 10A-B). Using CTRP drug sensitivity testing, the researchers found that CCR1/2/3/4/5/6/7/9/10 were resistant to multiple drugs (Fig. 10A), such as sotrastaurin, belinostat, and LRRK2-IN-1. According to GDSC drug sensitivity tests, they found that CCR1/2/3/4/5/6/7/8/9/10 were resistant to various drugs (Fig. 10B), such as 5-Fluorouracil, I-BET-762, and Vorinostat.

## Discussion

GC can severely affect the health and lives of people across the globe, owing to its high morbidity and mortality rates [33–35]. A majority of the patients do not show any early symptoms of GC, while some display nonspecific upper gastrointestinal symptoms that resemble gastric ulcers. The most recent National Comprehensive Cancer Network guidelines place more emphasis on precision therapies, especially prognostic biomarkers, drug targets, and immune-related genes, due to the advancements in biomedical detection technologies and a better comprehension of the GC-related immune microenvironment [36].

There are 10 CC chemokine receptor subtypes, making them the largest subdivision in the chemokine superfamily. All ten are G protein-coupled receptors with a 7-transmembrane region, and they primarily trigger the signal transduction pathway through the Gi proteins [22]. A collection of genes on chromosome 3p21 in humans encode the proteins CCR1, CCR2, CCR3, CCR4, CCR5, CCR8, CCR9, and CCR10. A gene on the long Chromosome 6 arm (6q27) codes for CCR6, and a gene on the Chromosome 17q21.2 codes for CCR7. CCRs mediate different forms of immunological responses and are significantly and differently expressed on many of the leukocyte subsets [37]. They play a role in many inflammatory and autoimmune diseases, including osteoarthritis, inflammatory bowel disease, and multiple sclerosis [21, 38–40]. Several studies have highlighted the significant role played by the CC chemokine receptors in mediating the chronic inflammatory response, as well as leukocyte recruitment, angiogenesis, and metastasis of tumors [41, 42]. The CC chemokine receptors can be regarded as a potential pharmacological target [22]. A few CC chemokine receptor antagonists and/or inhibitors have exhibited remarkable anti-tumor efficacy in preclinical studies and clinical trials [41, 43]. However, none of the researchers, to date, have characterized the prognostic values and the biological roles of the CC chemokine receptors in GC patients.

In this study, the researchers studied the CC chemokine receptor expression level in GC patients and discovered that six genes were expressed differentially in gastric tumor tissues in comparison to the normal tissues (the results indicated that CCR1, CCR4, CCR5, and CCR8 genes were upregulated, whereas the CCR6 and CCR10 genes were downregulated). They also examined the prognostic significance of the CC chemokine receptors in GC patients. The findings demonstrated a substantial correlation between low OS rate and the higher expression levels of genes like CCR4, CCR5, CCR7, CCR8, CCR9, and CCR10 and the lower expression levels of CCR3 and CCR6. The low expression levels of CCR3 and CCR6 were significantly related to a worse FPS, whereas the high expression levels of CCR8, CCR9, and CCR10 led to a poor FPS in GC patients. With regard to the PPS, a higher expression level of the CCR1, CCR2, CCR5, CCR7, CCR8, CCR9, and CCR10 genes and a lower expression level of the CCR3 and CCR6 genes were seen to be significantly related to a worse PPS. In summary, it shows that CCR4, CCR5, CCR8, and CCR10 are harmful factors for humans and CCR6 is a protective factor. Previous research has shown that CCR4 and its ligands were associated with increased tumor recurrence and impaired OS in patients with GC [44]. CCR5 expression was associated with lymph node metastasis and a worse prognosis in patients with GC and shown to be an independent indicator of a poor prognosis in GC [45]. CCR6 expression is deregulated in some human malignancies and may be involved in the tumor progression. zhang et al. showed that CCR6 was highly expressed in GC tissues and was a factor for poor prognosis, which is different from what we found in our study, probably due to different selection of specimens, and more samples are needed to clarify [46]. An upregulated expression of CCR8 in GC tissues was associated with tumor grade, nodal metastasis, and OS. Tumor-infiltrated Tregs with higher expression of CCR8 produced more IL10 molecules in vitro [47]. No studies of CCR10 in GC at this time.

And for the prognostic value of the DNA methylation of CC chemokine receptors 4 CpGs of CCR1, 1 CpG of CCR2, 1 CpG of CCR3, 4 CpGs of CCR4, 2 CpGs of CCR5, 2 CpGs of CCR6, 1 CpG of CCR7, 1 CpG of CCR8, 3 CpGs of CCR9 and 1 CpG of CCR10 were significantly associated with prognosis in GC patients. Furthermore, the researchers also studied the molecular properties of the CC chemokine receptors in GC patients. The onset and progression of GC is a multi-step multi-factor process that also includes genetic variations. Hence, in this study, the researchers investigated the genetic alterations that took place in the GC patients compared to the normal samples. Their results revealed that the CCR genes in the GC patients showed a 0.7-4% genetic variation. They used the GeneMANIA software and noted that the networks associated with these CCRs were mainly associated with the shared protein domains, physical interactions, co-expression, predicted, and co-localization.

A few researchers noted that the presence of the TILs in the GC patients was related to better prognosis; additionally, it could be regarded as an indicator of an effective immune response against the tumors [48]. In this study, the researchers determined the relationship between the CCRs and the infiltration of the different kinds of immune cells in GC patients. They noted that the expression levels of the 10 CCRs were positively linked to the infiltration of 6 different immune cells, except for CCR2 and CCR10 in B cells. The researchers used single-cell analysis to further elucidate the role of the expression levels of the CCRs in the tumor-infiltrating immune cells in GC and observed that the T-cells and the macrophages were highly expressed in the CCRs when the CCR10 expression was high in plasma cells.

Finally, The researchers evaluated drug-sensitive CC chemokine receptors in GC and found that no sensitive drugs are currently available. However, insensitive drugs, such as 5-Fluorouracil, can be avoided in clinical practice.

Our results suggest that some CC chemokine receptor variants are deleterious for GC patients. Thus, the researchers can predict the prognosis and evaluate the immune microenvironment by detecting the expression level of the CC chemokine receptors in GC patients. Therefore, the relevance of CC chemokine receptors in immune system function might explain why the presence of high CC chemokine receptors is an unfavorable prognostic factor in patients with GC. CC chemokine receptors are a potentially novel and valuable biomarker in GC.

However, there may be some restrictions on this study. To identify the relationships between various immune cells and the CC chemokine receptors, sequencing information from public libraries was examined. Therefore, additional experimental validation is required. In addition, as a promising prognostic predictor and prospective immunotherapy target, the possible effects and mechanisms of CC chemokine receptors in GC merit further investigation.

## Data Availability

The datasets generated and/or analysed during the current study are available in the Human Protein Atlas (HPA) (https://www.proteinatlas.org/), Kaplan–Meier plotter(http://www.kmplot.com), GeneMANIA(http://www.genemania.org), MethSurv(https://biit.cs.ut.ee/methsurv/), UALCAN(http://ualcan.path.uab.edu/), GSCA(http://bioinfo.life.hust.edu.cn/GSCA/#/), cBioportal(http://cbioportal.org), and TIMER(https://cistrome.shinyapps.io/timer/).

## Abbreviations

TME: Tumor microenvironment
GC: Gastric cancer
HPA: Human Protein Atlas
KM: Kaplan-Meier
UALCAN: University of ALabama at Birmingham CANcer
GSCA: Gene Set Cancer Analysis
TIMER: Tumor IMmune Estimation Resource
TILs: Tumor-infiltrating lymphocytes
OS: Overall survival
scRNAseq: single-cell RNA sequence
PBMCs: Peripheral Blood Mononuclear Cells
TCGA: The Cancer Genome Atlas
GDSC: Genomics of Drug Sensitivity in Cancer
CTRP: Cancer Therapeutics Response Portal
FPS: First-progression survival
PPS: Post-progression survival
